# Water Molecular System Dynamics Associated with Amyloidogenic Nucleation as Revealed by Real Time Near Infrared Spectroscopy and Aquaphotomics

**DOI:** 10.1371/journal.pone.0101997

**Published:** 2014-07-11

**Authors:** Eri Chatani, Yutaro Tsuchisaka, Yuki Masuda, Roumiana Tsenkova

**Affiliations:** 1 Department of Chemistry, Graduate School of Science, Kobe University, Kobe, Hyogo, Japan; 2 Department of Environmental Information and Bioproduction Engineering, Graduate School of Agricultural Science, Kobe University, Kobe, Hyogo, Japan; King’s College, London, United Kingdom

## Abstract

The formation of amyloid fibrils proceeds via a nucleation-dependent mechanism in which nucleation phase is generally associated with a high free energy resulting in the rate-limiting step. On the basis of this kinetic feature, the nucleation is one of the most crucial phases controlling the pathogenesis of amyloidoses, but little is known about the details of how protein molecules and surrounding environment vary at this stage. Here, we applied near infrared (NIR) spectral monitoring of water structural changes in real time during the nucleation-dependent fibrillation of insulin. Whilst multivariate spectral analysis in the 2050–2350 nm spectral region indicated cross-β formation, characteristic transformations of water structure have been detected in the spectral region 1300–1600 nm corresponding to the first overtone of water OH stretching vibrations. Furthermore, specific water spectral patterns (aquagrams) related to different water molecular conformations have been found along the course of protein nucleation and aggregation. Right in the beginning, dissociation of hydrogen-bonded network in bulk water and coinstantaneous protein and ion hydration were observed, followed by water hydrogen-bonded networks development, presumably forcing the nucleation. These specific transformations of water spectral pattern could be used further as a biomarker for early non-invasive diagnosis of amyloidoses prior to explosive amplification and deposits of amyloid fibrils.

## Introduction

Amyloid fibrils are supramolecular protein self-assemblies associated with amyloidoses [1]–[3]. Clarification of the mechanism underlying the formation of amyloid fibrils has been a crucial subject indispensable for diagnosis, treatment and prevention of diseases. The fibril formation generally proceeds via a nucleation-dependent mechanism, and a wealth of reports has suggested that nucleation phase involves a high energetic barrier that limits the rate of the overall reaction [4]–[7]. Considering this energetic property, exploring detailed mechanism of nucleation is one of the most essential issues that still require clarification for understanding of the pathogenesis. However, the lag phase is often monotonous without any prominent protein spectroscopic or thermodynamic signals, and much remains to be elucidated regarding how and when nuclei species which template for fibril growth emerge; although oligomer-like intermediates have been captured successfully for some proteins [8]–[11], their characterization is often difficult due to low population and details of nucleation are still poorly understood, only with atomic images of protein assembly to form oligomeric intermediates as proposed by computational researches [12], [13].

To explore this issue, we postulate that the molecular structure of water surrounding proteins in aqueous systems will provide valuable clues as to detailed mechanisms of nucleation. Water is the most abundant component of biological systems and considered to work closely with the solute proteins by binding or interacting with them, contributing to protein folding, dynamics, and functionality [14]. In this study, we have applied near infrared (NIR) spectroscopy to monitor water structural changes in real time during the fibrillation reaction of human insulin, a 51-residue hormone protein consisting of two polypeptide chains associated with regulating glucose metabolism. Non-invasive NIR spectroscopy has been widely applied since 1980s for evaluating the quality of agricultural and pharmaceutical products [15] and its use is expanded to water structural analyses [16], [17]. Recently, it has been found that distinct water conformations with fundamental molecular vibrations located in the mid infrared region, e.g., dimers, trimers, solvation shells etc., have specific spectral patterns as overtones and combination bands in the NIR region where non-invasive monitoring can be done using longer pathlength. Therefore, NIR spectroscopy is becoming a powerful analytical technique for diagnosis and for investigation of the rearrangement dynamics of the water molecular network [17].

As for amyloid fibrils, there are several reports for the interaction between protein and water molecules such as water-filling inside the amyloid fibril structure [18] and hydration or the transient organization of fibril-water network [19]–[22]. Furthermore, we have successfully monitored difference in hydration state of prion protein isomers dependent on the type of metal ions by using the NIR technique, which has suggested specific water structure associated with potent amyloidogenicity [22]. Recently, a novel concept of “aquaphotomics” has been proposed to focus attention on water molecular system as a potential source of information playing a key role as “water molecular mirror” to be further used as biomarker for health diagnosis of living systems [16], [17]. According to the aquaphotomics viewpoint, water structural changes are easily detectable with near infrared light as specific spectral patterns, aquagrams [16]. Here, we have shown unique transformations of water spectral patterns, suggesting a transient water molecular dissociation leading to protein and ion hydration and subsequent organization of hydrogen-bonded water networks militating the nucleation phase of insulin fibril formation.

## Materials and Methods

### Materials

Recombinant human insulin was obtained from Wako Pure chemical Industries, Ltd. Concentrations of insulin were determined using an absorption coefficient of 1.0 for 1.0 mg/ml at 276 nm [23].

### Near-infrared spectral monitoring of fibril formation

The spontaneous formation of insulin amyloid fibrils without seeds was performed by heating under acidic conditions [24]–[26]: the human insulin dissolved in 25 mM HCl containing 100 mM NaCl at a concentration of 3.0 mg/ml was used as a sample. The fibrillation reaction was initiated by jumping temperature of the protein solution sealed inside a quartz liquid sample cell with a 1-mm optical pathlength to 75°C, and then it was kept constant until the completion of the NIR measurement. NIR transmission spectral monitoring has been done for 30 min in the range of 400–2500 nm with a step size of 2 nm by NIR spectrophotometer (MPA, Bruker Optics, Germany). Spectral data contained spectra acquired at every minute and calculated as averages of 3 consecutively measured spectra. For the partial least squares (PLS) analysis, samples at a concentration of 0.5, 1.0, and 5.0 mg/ml were additionally measured.

### Multivariate analysis

As the absorbance spectra of the first overtone of water OH stretching vibrations in the NIR spectral region contains a multitude of various bands, multivariate spectral data analysis methods like principal component analysis (PCA) commonly used so far has been applied for unraveling the immense information in the spectra. In this study, all multivariate spectral analysis was carried out by MATLAB (The MathWorks, Inc., Natick, MA, USA) software program. PCA was used for data compression by using orthogonal matrix decomposition, in which principal components are orthogonal to each other and define a pattern space which explains all the variation in the data. As the absorbance reached plateau within 30 min, the data from 1 min to 30 min were selected for analysis thereafter. For the analysis in the protein region of 2050–2350 nm, standard normal variate (SNV) correction was applied to the spectra prior to PCA in order to compensate for scattering derived from fibril formation. This approach successfully cancelled the time-dependent change in baseline absorbance caused by size development of protein assemblies during fibrillation. On the other hand, it should be noted that, for the PCA of the spectral data in the first overtone of the water region, 1300–1600 nm region, pretreatment by SNV correction was NOT performed because it caused distortion of the PC component related to water structural changes. The relationship between actual and predicted concentrations of insulin protein was examined by PLS regression, based on leave-one-out cross validation. In the present analysis, the informative absorbance bands with high intensity in the regression vector were identified by using 6–10 min and the 1–60 min datasets as calibration and prediction sets, respectively. After these analyses, obtained PCA loadings and PLS regression vectors were smoothed using Savitzky-Golay second derivative polynomial filter with window size of 12 to facilitate the determination of peak positions.

### Construction of aquagram

To identify the wavelength range related to fibril formation, a radar chart named aquagram was constructed to visualize water spectral pattern at different time points. Fourteen characteristic water absorbance bands (wavelengths) which cover most distinctive species of water structure termed water matrix coordinates (WAMACS) were used for axes and the values for aquagram were obtained according to the following equation;
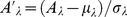
where *A_λ_*, *µ_λ_*, and *σ_λ_* are absorbance after multiplicative scatter correction (MSC), mean of all spectra, standard deviation of all spectra, respectively, at wavelength *λ*.

### Atomic force microscopy (AFM)

Ten-fold diluted by water 10 µl fibril samples were spotted onto a freshly cleaved mica plate. After 2 minute, the residual solution was removed by placing a piece of filter paper at the edge of the mica plate and then dried. AFM images were obtained using a NX10 (Park Systems). The micro cantilever used was a phosphorus (n)-doped Si (Olympus, spring constant = 18.5–34.6 N/m, resonant frequency = 264.9–332.5 kHz), and the scan rate was 0.5 Hz.

### Fourier transform infrared (FTIR) absorption measurement

Attenuated total reflectance-FTIR spectra were measured with a J-6100 model spectrometer (Jasco, Japan) with an ATR option. Amyloid fibrils formed (2 µl) were loaded and dried on the prism for the measurement. FTIR spectra were monitored at room temperature by collecting 256 interferograms with a resolution of 2 cm^−1^.

## Results

### NIR spectral measurement during the heat-induced spontaneous fibrillation

**Figure 1:**
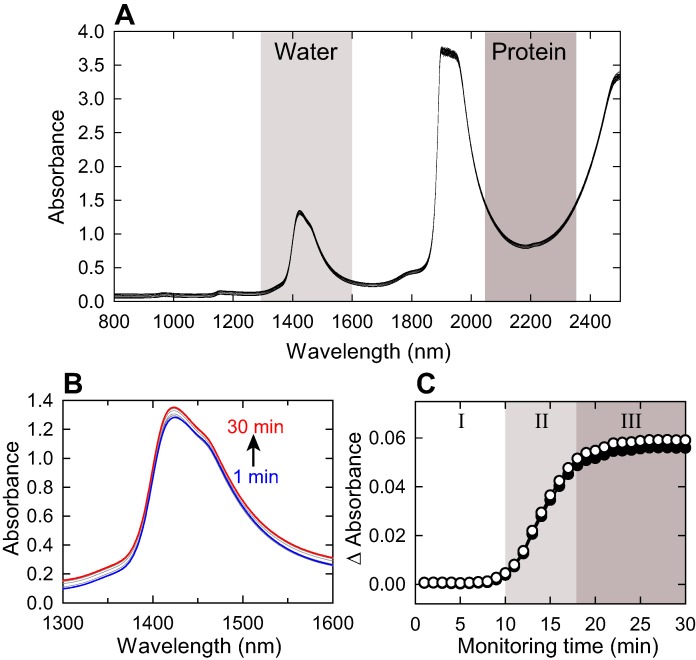
NIR spectra of insulin solution monitored in the present study. (A) Raw NIR spectra data set. Two regions over 1300–1600 nm and 2050–2350 nm were focused on to evaluate changes with water and protein structures, respectively. (B) A magnified view at 1300–1600 nm. The direction of absorbance increment is indicated by an arrow. (C) Time-dependency of absorbance. Absorbance increment obtained by averaging absorbance over 1300–1600 nm (closed circles) or 2050–2350 nm (open circles) was plotted against time. The three phases, i.e., phases I (nucleation), II (elongation), and III (equilibrium), are represented.

We have performed *in-situ* NIR spectral monitoring of the heat-induced fibrillation reaction of human-derived insulin protein [24], [25] dissolved in 25 mM HCl containing 100 mM NaCl at constant temperature 75°C. The spectra of the sample solution showed two strong water absorbance bands at around 1450 and 1950 nm (Figure 1A). As the latter band is too strong and saturated under the present measurement conditions, the region of the first overtone of water stretching vibrations, 1300–1600 nm, was focused on to analyze water structural changes in this work. Additionally, the region of 2050–2350 nm which includes combination bands related to hydrogen bonds of amide groups and is thus sensitive to the secondary structure of proteins [27]–[30] was also analyzed to monitor structural change of proteins occurring concurrently with water structures during the fibrillation reaction.

Upon heating for the initiation of fibrillation, slight but statistically significant increase in baseline absorbance was observed approximately 10 min after starting the reaction as well as the measurement (Figure 1B). When increment of absorbance baseline was calculated by averaging absorbance over 1300–1600 nm and 2050–2350 nm regions at each time point and plotted against monitoring time, according to the following equation;
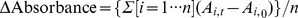
where *A_i,t_* and *A_i,_*_0_ represent absorbance of *i*th data point at time *t* and initial time of measurement (i.e., 1 min), respectively, an abrupt increase in absorbance baseline was observed after a 10-min lag time, consistent with the spontaneous formation of amyloid fibrils consisting of nucleation and growth phases (Figure 1C). Based on the fitting of this light scattering intensity with a sigmoidal curve according to a previous report [23], the fibrillation time course was categorized into three phases, i.e., phases I (1–10 min), II (10–18 min), and III (18–30 min), which are referred to as nucleation, elongation, and equilibrium phases, respectively [5], [31].

### Tracking of cross-β formation by the spectral change over 2050–2350 nm

To further investigate the transformation of protein structures during the fibrillation reaction, PCA has been performed in the 2050–2350 nm spectral region. In this region, an overtone of combination bands of the free NH stretching-amide II (amide A/II) and intramolecular hydrogen-bonded NH stretching-amide II (amide B/II), and CH stretch/CH deformation appear, and therefore, the spectral analysis of the corresponding region is applicable to exploring the structural changes of proteins [29], [30].

**Figure 2:**
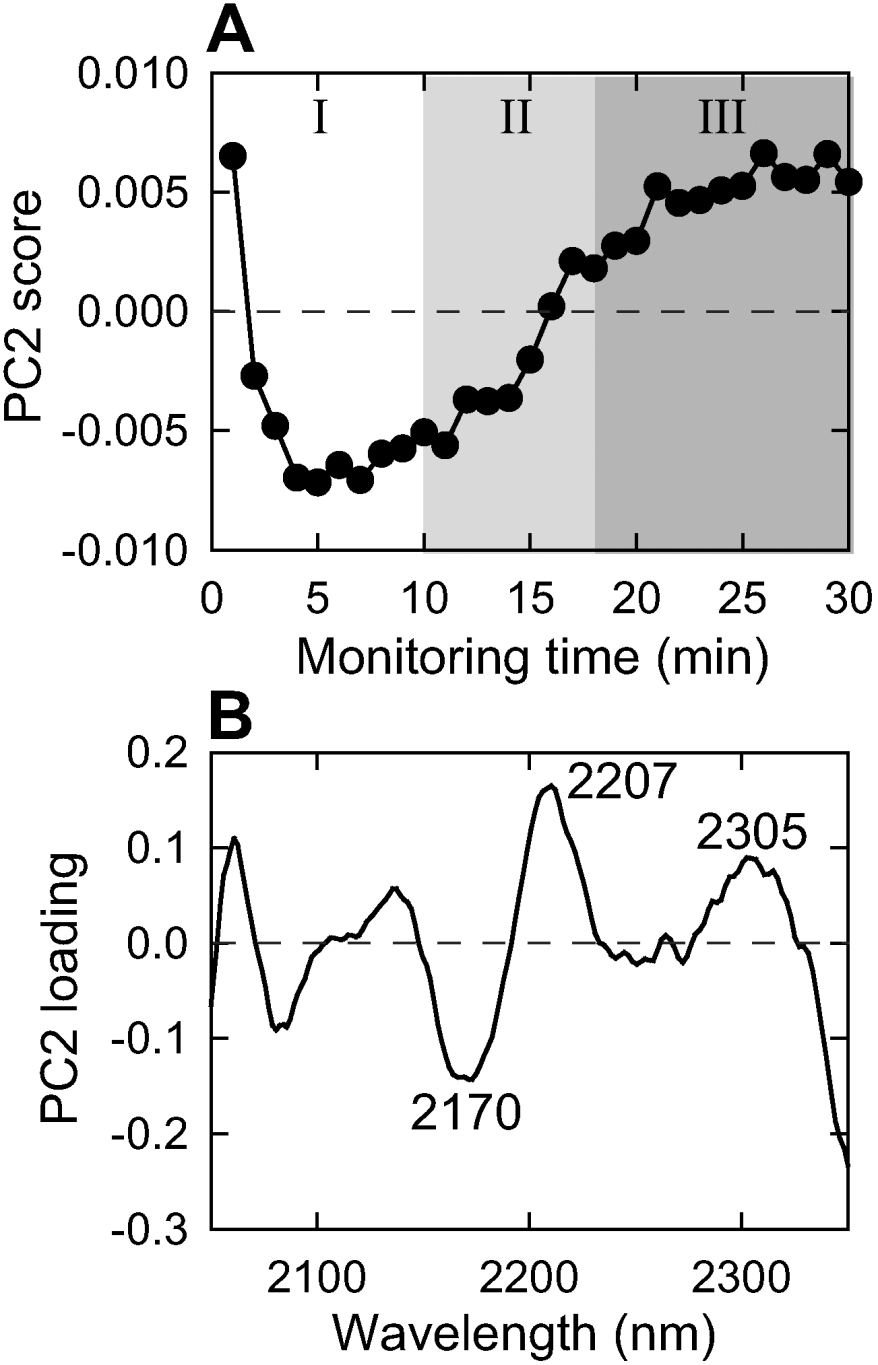
PCA of spectral data acquired at amide I overtone region for the fibril formation of insulin indicating α-helix to β-sheet transition during the formation of amyloid fibrils. (A, B) PC2 score plot with monitoring time (A) and its loading (B). The variation of PC2 was 0.463% and the results of other PC components are shown in Figure S1.

As a result, we have observed a characteristic behavior for PC2, where the score values decreased immediately after the initiation of the reaction and after 10 min, increased again and finally saturated approximately after 20 min (Figure 2A): the shape seemed fundamentally similar to the change in light scattering. Although the strict assignment was still difficult because of complicated appearance of combination bands, PC2 loading spectral pattern showed several peaks related to α-helix (2170 nm) and β-sheet (2207 and 2305 nm) [27], [29], [32], from which the cross-β formation in conjunction with the prompt increase in light scattering has been clarified (Figure 2B). For PC1, no characteristic peaks were observed and its time dependency seemed to be completed within 5 min, suggesting change in background intensities accompanying the initial temperature jump (Figure S1 A and C). For PC3, although one negative peak was found at 2336 nm, its scores fluctuated randomly throughout the measurement time period and it was therefore difficult to find any correlations with nucleation or elongation phases of the fibril formation (Figure S1 B and D). The transformation of protein structure was also investigated by partial least squares (PLS) regression analysis, which also supported the abrupt increase in cross-β structures after undergoing the lag phase for nucleation (Figure S2).

**Figure 3:**
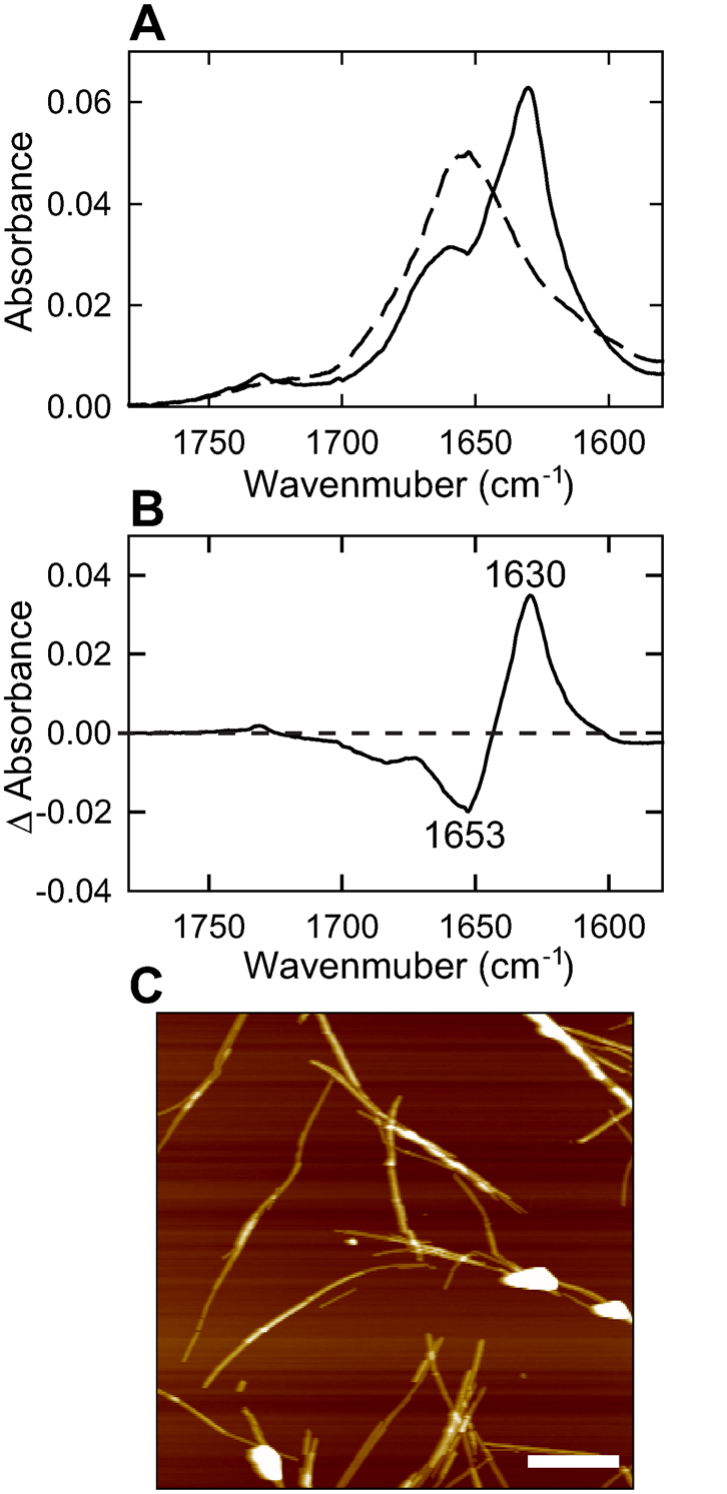
Amyloid fibrils formed inside the optical cell after the NIR measurement confirmed by FTIR and AFM measurements. (A) FTIR absorption spectrum at around the amide I region (solid line). The dashed line represents spectrum of intact insulin without any heating treatment as a reference. The spectra were normalized so that the integrated intensity of the amide I band ranging from 1580 to 1780 cm^−1^ is set to be equal. (B) Difference FTIR spectrum plotted by subtracting the spectrum of sample solution after the NIR measurement (A, solid line) from that of intact insulin (A, dashed line). (C) AFM image. The scale bar inside the image represents 1 µm.

To verify the formation of amyloid fibrils inside the optical cell, the fibrils sampled after the NIR measurement were subjected to FTIR and AFM analyses. The obtained FTIR absorption spectrum revealed a marked difference in shape on amide I region from that obtained by the protein solution without heating (Figure 3A), and the difference spectrum exhibited significant positive and negative peaks at 1630 cm^−1^ and 1653 cm^−1^, respectively (Figure 3B), demonstrating the structural transformation from α-helix rich native conformation to cross-β structure. AFM images also demonstrated the formation of amyloid fibrils with needle-like morphology inside the optical cell (Figure 3C).

### Analysis of water structural changes observed by the spectral changes over the water 1st overtone 1300–1600 nm region

**Figure 4:**
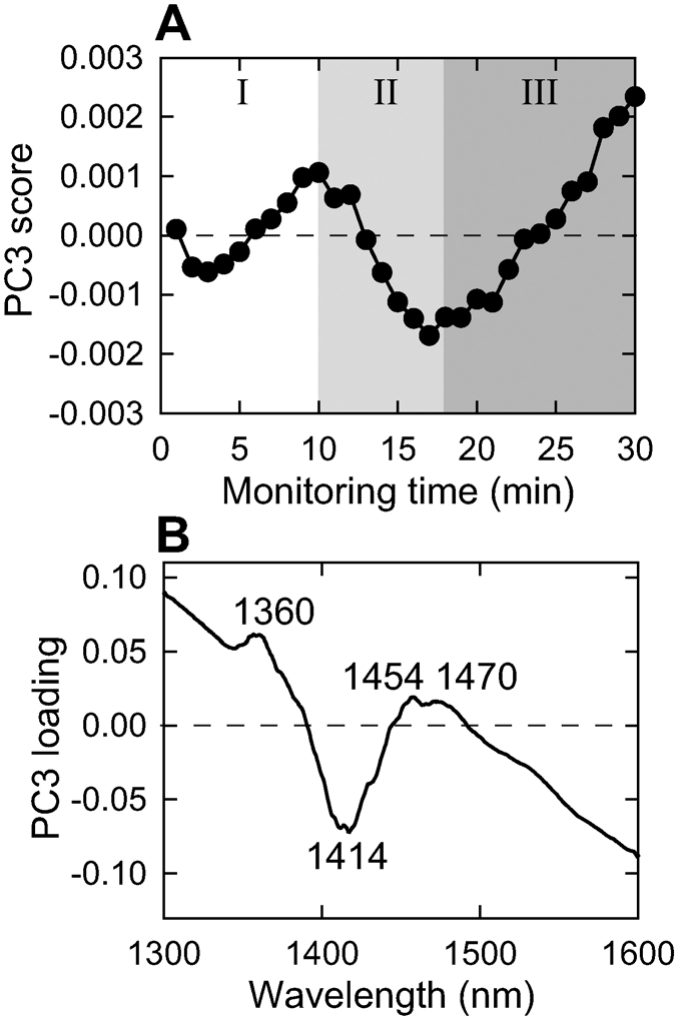
PCA of spectral data acquired at water first overtone region for the fibril formation of insulin indicating conformational changes of water molecules. (A, B) PC3 score plot with monitoring time (A) and its loading (B). The variation of PC3 was 0.0005% and the results of other PC components are shown in Figure S3. PC3 loading presents interplay of opposite spectral changes of hydrogen bonded water species (1360 nm, 1454 nm, and 1470 nm) and water molecular species with free hydrogen bonds (1414 nm) along the examined process of insulin fibril formation.

To monitor water molecular structures simultaneously with protein structures in real time during the fibrillation reaction, PCA of insulin solution spectral data has been performed in the spectral region 1300–1600 nm corresponding to the first overtone of water OH bonds. As a result, we have found characteristic transformations of water structure depicted by the spectral pattern of the PC3 loading. The PC3 score plot (Figure 4A) exhibited changes in a zig-zag manner, and the time course of each change coincided well with the proposed nucleation, elongation, and equilibrium phases. For PC1 and PC2, the score changes were attributed mainly to change in the light scattering accompanying the fibrillation reaction, and to that accompanying initial temperature jump of the sample solution (Figure S3). The loading of PC3 showed a spectral pattern with notable negative peak at 1414 nm characteristic of the free water (S_0_), and additionally, several positive peaks characteristic for water solvation shell (1360 nm) and hydrogen-bonded OH (1454 nm and 1470 nm, which are assigned to intermolecularly hydrogen-bonded hydroxyls and S_3_, respectively), where S_n_ represents the number of hydrogen bonds (i.e. S_0_ for free water molecular species without any hydrogen bonds for instance) (Figure 4B) [17]. Furthermore, the loading presented a significant slope in baseline decreasing towards longer wavelengths, which is assumedly attributed to the decrease in amount of bulk water molecules [15] over the measurement. For a control, the PC scores and loadings of the solvent (i.e. 25 mM HCl containing 100 mM NaCl) were also analyzed, but they did not reveal any similarities to the PC3 of the insulin sample, except for the decrease in the slope (Figure S4). Moreover, when similar analysis was performed at lower concentration of insulin (1 mg/ml) and in the absence of NaCl, where fibril formation did not occur within 60 min, PCA result did not show any components (data not shown), supporting that the above PC3 represents transformation of water structures in association with the formation of amyloid fibrils.

When the directions of the peaks in the loading and the change in score are considered together, the multi-step changes in the PC3 score indicate a dynamic formation and destruction of hydrogen-bond-network water conformations (Figure 4). It is especially of note that significant score changes were monitored during the nucleation phase where light scattering and protein secondary structure didn’t exhibit any significant signals. The scores mainly increased in upward direction, indicating that the cluster-like water structures including solvation shell and hydrogen-bonded species were formed, and alternatively, free water species decreased relatively in the nucleation process (Figure 4). Intriguingly, a slight decrease in the PC scores preceded its positive change, from which two-step structural arrangements of water molecules have been discovered for the nucleation. The formed hydrogen-bonded water clusters seemed to disappear along with abrupt amplification of fibrils during the elongation phase, and again developed after reaching the equilibrium phase (Figure 4A). Repeated measurement of the same reaction at different experiments verified that the spectral change showed reproducibility, suggesting that the time-dependent behavior observed for PC3 represents the changes in the protein–water interactions due to changes in protein structure during the formation of amyloid fibrils (Figure S5). The structural transformation of water molecules was further investigated by PLS regression analysis of insulin concentration, supporting the PCA results (Figure S6).

### Characterization of the transformation of water structures by aquagram

**Figure 5:**
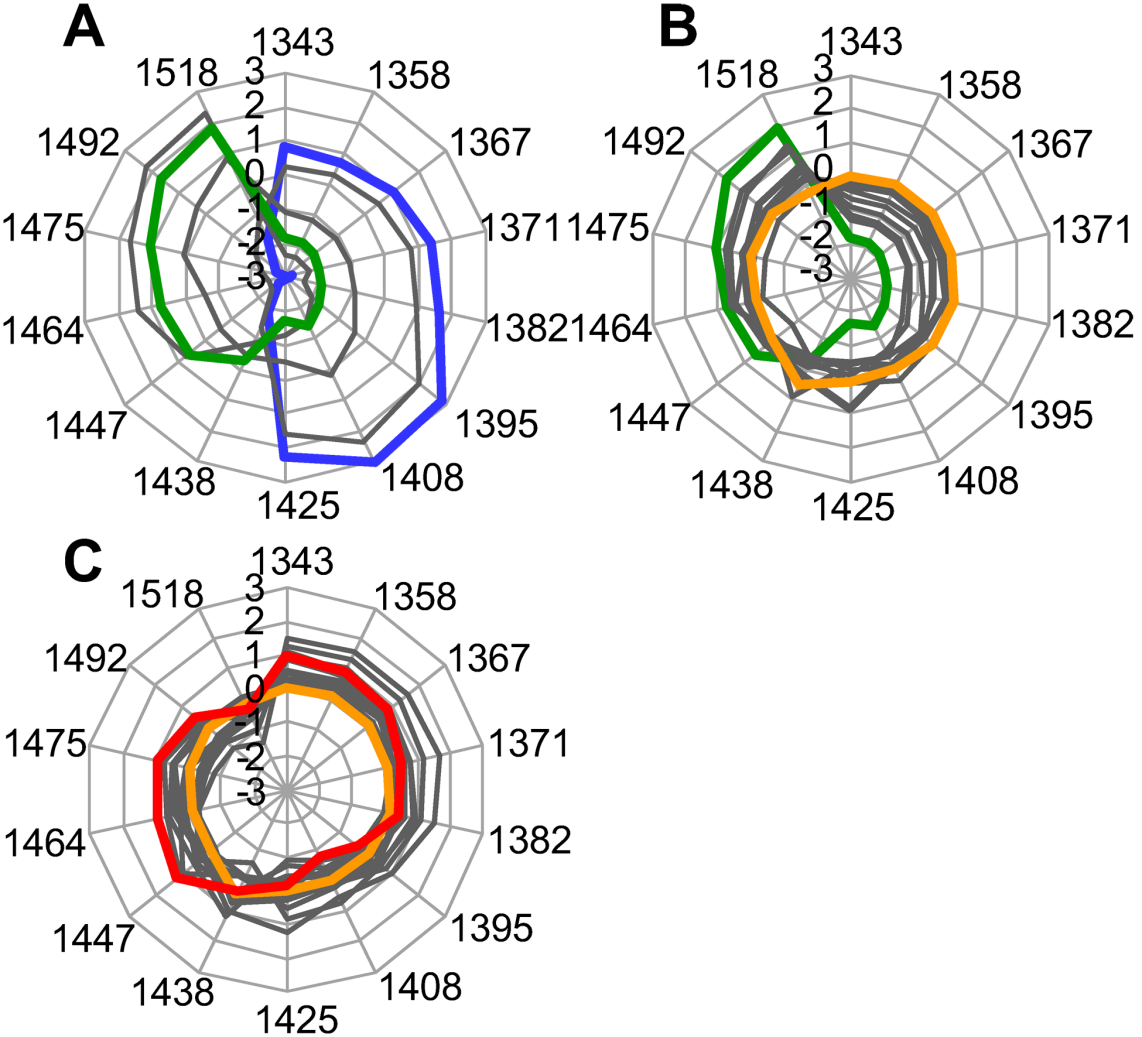
Time dependency of water spectral changes along the fibril formation process depicted by aquagram patterns. (A) 6 to 10 min for nucleation. (B) 10 to 18 min for elongation. (C) 18 to 30 min for the equilibrium phase. The normalized by the respective standard deviation value of the spectral difference between each absorbance and the average for the whole data set is represented starting from the center of the graph (the point 0). The water absorbance pattern is plotted every 1 min, starting from the 6th minute, and those at 6 min, 10 min, 18 min, and 30 min are colored by blue, green, orange, and red, respectively. The rest are colored by gray.

To investigate detailed characteristics of water structural transformation occurring in connection with the fibril formation, especially nucleation, the obtained NIR spectra were subjected to analysis of the absorbance pattern at specific water bands, which we term as Water Matrix Coordinates (WAMACS); in previous studies, Tsenkova *et al.* have defined 14 characteristic water wavelength ranges which cover various conformations of water molecules and are thus useful to depict characteristic spectral pattern in the first overtone region of water [16]. The aqueous system of insulin under the perturbation of temperature and light when acquiring spectra over the time has shown the following activated water absorbance bands; 1343 nm (ν_3_ of H^+^·(H_2_O)_3_), 1358 nm (H^+^·(H_2_O)_8_), 1367 nm (ν_1_ and/or OH^−^·(H_2_O)), 1371 nm (H^+^·(H_2_O)_5_), 1382 nm (OH^−^·(H_2_O)_5_ and/or O_2_^−^·(H_2_O)_4_), 1395 nm (dehydrating water), 1408 nm (ion hydration, i.e., OH stretch of O-H···O and/or S_0_), 1425 nm (protein hydration), 1438 nm (H^+^·(H_2_O)_2_) 1447 nm (S_1_), 1464 nm (S_2_), 1475 nm (S_3_), 1492 nm (S_4_), 1518 nm (ν_1_, ν_2_). For these assignments, ν_n_ represents OH stretching vibrations of hydrogen-bonded water molecules (i.e., ν_1_; symmetric stretching, ν_2_; bending, and ν_3_; asymmetric stretching) [17], [33]–[37]. For each wavelength range, mean-centering and normalization of dispersion was conducted for the construction of aquagram, a radar chart displaying time-dependency of normalized absorbance at these water bands which are represented on radar axes (Figure 5) [16].

As a result, although data of 1–5 min had to be excluded for this analysis because the NIR spectrum was strongly influenced by temperature change of sample solution as represented by the PC2 score in PCA analysis, the aquagram exhibited quite a characteristic water absorbance pattern biased towards 1382, 1395, 1408, and 1425 nm at 6 min. It suggests dissociation of hydrogen-bonded water clusters (S_2_, S_3_, and S_4_) followed by generation of free or weakly hydrogen-bonded water molecular structures involved in dehydration (1395 nm) and further on ion hydration (1408 nm), and protein hydration (1425 nm) (blue line in Figure 5). As time advanced, the free OH or O-H···O decreased and alternatively strong hydrogen-bonded water molecular structures, especially in the region of 1447 to 1518 nm appeared (green line in Figure 5), demonstrating that hydrogen-bonded network of water structures like S_2_, S_3_, S_4_ are re-organized, in good agreement with the two-step transformation of water structures for the nucleation phase as suggested by the PC3 scores of PCA analysis. These cluster-like water structures were reduced gradually through the fibrils growth phase, and the biased pattern of WAMACS was gradually altered towards a concentric pattern (orange line in Figure 5), though, a substantial difference was observed in hydration structures: for the insulin containing sample, a largest value was observed at 1438 nm, while for the control, 1395 nm showed a maximum. The final pattern after reaching the equilibrium phase seemed somewhat analogous to that observed for solvent (red lines in Figure 5 and Figure S7 for protein sample and solvent, respectively). Although constant NIR irradiation during the measurement seemed to influence water structures to a certain degree as seen in Figure S7, as suggested by the previous report [22], the water spectral pattern transition observed for protein sample was repeatedly different from that for solvent (Figure 5 and Figure S7), suggesting that the water structural change monitored in the present work is mainly caused by fibrillation of insulin.

## Discussion

The role of water molecules in amyloid formation has been explored mainly by means of computer simulation [38] and experimental strategy for direct observation of water structures has been restricted to several techniques such as magnetic resonance spectroscopy [39], [40] and calorimetric analysis [20], [41] because of fundamental difficulty in detecting dynamics of water molecules. This time, we have applied near infrared spectroscopy and aquaphotomics method to examine non-invasively time-resolved behavior of the whole process of fibrillation of insulin. In the NIR region, a large number of overtone and combination bands are severely overlapped, but the use of analytical methods, i.e., multivariate analysis [22], [42] and two-dimensional correlation analysis [27] has successfully separated each band from complicated spectrum leading to the application of the NIR spectroscopy for investigation of aqueous systems. In this work, multivariate analysis technique (PCA and PLS) was employed and as a result, although several principal components that exhibit the strong influence of the light scattering and temperature change constitute the majority of the entire variation, we extracted a reliable and reproducible principal component that describes the spectral change of water molecules (Figure 4). It was further supported by additional analytical methodology of PLS regression performed with a dataset of NIR spectra monitored at four different concentrations of insulin protein (Figure S6). Intriguingly, time-dependent changes of the scores of PC3 component showed several distinct stages (Figure 4A), the time scale of which mainly corresponded well with the nucleation, elongation, and equilibrium phases. It is conceived that structural changes plus mutual assembling of proteins to form oligomeric and/or supramolecular assemblies have considerable impacts transferred to surrounding water by interplaying with each other.

**Figure 6:**
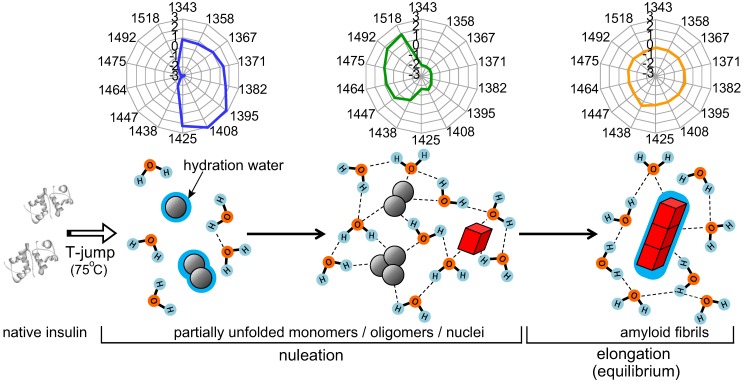
Schematic illustration representing multi-step transformation of water structures during the fibril formation. In the nucleation phase, free water molecules, free OH and protein hydrating water molecular species were initially dominating, but afterwards hydrogen-bonded water networks were developed, which was considered essential for nucleation by interlinking protein molecules. In the elongation phase, the hydrogen bonds were decayed gradually towards the state observed in bulk water, and slight increase of hydrating water onto amyloid fibrils was also observed. The aquagram patterns at 6, 10, 18 min are also represented at corresponding stages of the fibril formation.

The most explicit transformation of water structure clarified in the present work is the dynamic variation in the water hydrogen-bonded network during the nucleation phase. After the temperature settled down, in the beginning of the fibrillation spectral monitoring time series, a brief decrease of the absolute value of PC3 negative scores (Figure 4A) and the characteristic pattern of aquagram at 6 min (blue plot in Figure 5A) indicate that some amount of hydrogen-bonded water structures are decomposed initially resulting in the dominance of less hydrogen bonded water structures. Dehydration band, 1395 nm, and ionic and protein hydration bands, 1408 nm and 1425 nm, showed comparatively high absorbance. Thereafter, hydrogen-bonded water structure was organized to form cluster-like structures of water molecules through the rest of the nucleation phase, as indicated by the score of PC3 (Figure 4A) along with the PLS analysis (Figure S6). At this stage, the evolution of unique hydrogen-bonded water structure has been further verified by a highly characteristic aquagram pattern with a fan-like shape (green plot in Figure 5A), suggesting large fraction of water species with two, three, or four hydrogen bonds, and alternatively low fractions of less hydrogen bonded water species. On the basis of the present investigation, a schematic model has been summarized for the transformation of water structures as represented in Figure 6.

There has been no experimental reports describing the development of such hydrogen-bonded water structures during the stage of nucleation phase, but intriguingly, a similar increase in water species making two hydrogen bonds (S_2_) in the early stage of the fibril formation was observed for the manganese-bound prion protein [22], although continued study on molecular vibration and careful consideration of effects of external perturbation including temperature and pH on each peak position is required to achieve more robust peak assignment and comparison. It has been proposed that the S_2_ water species is of special importance playing a role of activating water dynamics [22], [43]. If it is assumed that the organized hydrogen-bonded water network is not restricted within the proximal region but extended to considerably distal ones, it is likely that the water network contributes to interplay among an ensemble of protein molecules regulating well-ordered assembling. In light of a novel concept which has been recently proposed on the basis of explicit-water molecular dynamics simulations [13], water-mediated attraction between protein molecules contributes to directional adjustment towards a stable associated structure, which will lead to nucleation in the present case. After the nucleation, the hydrogen-bonded water conformations seemed to be attenuated along with abrupt amplification of fibrils during the elongation phase, and again developed after reaching the equilibrium phase.

From a series of investigations performed on insulin amyloid fibrils, we conclude that NIR spectroscopy is a powerful technique which should facilitate comprehensive understanding of fibrillation process, especially of the nucleation phase. The present result has demonstrated that the changes in the water absorbance pattern in the NIR region have enormous potential to reveal molecular aspects of how proteins and their ambient environment vary at each stage of fibrillation process. Through recent enthusiastic investigations, it is becoming evident that water is very sensitive to the conditions of biomacromolecules contained within the system and mirrors even minute changes, as seen by quantification of somatic cells in milk for diagnosis of cow’s mammary gland inflammation [44], detection of NIR spectral change for soybean plants infected with mosaic virus [45], wettability through the analysis of hydrogen bond properties of water molecules absorbed on oxide surfaces [46], and spectral pattern of urinary water as a biomarker for estrus of panda [47]. Likewise, the application of NIR measurement to amyloid study should open a new venue in the research field of protein misfolding and aggregation and furthermore, will lead to achievement of non-invasive diagnosis of amyloidoses as a unique indicator of an early sign of aberrant aggregation state of protein molecules at latent symptomless stage. The present investigation will further contribute to exploring a picture of microscopic interactions between water and protein molecules. Although there have still been only a small number of examples analyzing protein solutions, the studies of molten globule-like state of ovalbumin [27] and heat-induced denaturation of bovine serum albumin [42] revealed significant spectral changes in protein hydration state that are observed in conjunction with protein structural changes. Further accumulation of experimental data on water bands during various types of protein conformational changes including multimolecular association or dissociation process will provide valuable insights into detailed picture of protein organization and assembly, and furthermore, a molecular indication for the discrimination of aberrant state of water structures from biologically functional one.

## Supporting Information

Figure S1 **PC scores and loadings obtained as results of PCA at amide I overtone region in addition to PC2 (see** **Figure 2** **).** (A, B) PC1 (A) and PC3 (B) score plots with monitoring time. (C, D) Their loadings (PC1 (C), PC3 (D)). The variations for PC1 and PC3 were 99.5362% and 0.0003%, respectively. For PC1, No characteristic peaks were observed and its time dependency seemed to be completed within 5 min, suggesting change in background intensities accompanying the initial temperature jump. For PC3, although one negative peak was found at 2336 nm, its score fluctuated randomly throughout the measurement time period and it was therefore difficult to find any correlations with nucleation or elongation phases of the fibril formation.
(TIF)

Figure S2 **Spectral and structural changes of insulin protein molecules as estimated by PLS regression analysis.** The analysis was performed with a dataset of NIR spectra over 2050–2350 nm monitored at four different concentrations of proteins; 0.5, 1, 3, and 5 mg/ml. (A) Regression vector obtained as a model for the prediction of insulin concentration. The regression vector was based on the spectra at 6–10 min. A negative and positive peak at around at 2240 nm (α-helix) and 2300 nm (β-sheet) respectively, were observed, suggesting that conformational transition from α-helix to β-sheet. (B) Time-dependent change in the standard error of prediction (SEP) of the insulin concentration. In this analysis, the spectra at 6–10 min and 1–60 min were used for the model and test datasets, respectively. Although a marked larger value at 1–5 min might indicate mainly spectral change associated with temperature jump, the gradual increase in SEP values in the range of 11 min to 30 min coincides roughly with the elongation phase, verifying the formation of β-sheet rich fibril structure. (C) Time-dependent change of the ratio of absorbance at 2239 nm (assigned to α-helix) and that at 2205 nm (assigned to β-sheet). The result of protein solution is plotted by closed circles and that of solvent (25 mM HCl containing 100 mM NaCl) is also shown by open circles for reference. A marked decrease of the value in elongation (phase II) was observed, suggesting the abrupt increase in cross-β structure after undergoing the lag phase for nucleation, in accordance with the PLS result.
(TIF)

Figure S3 **Information of PC scores and loadings obtained as results of PCA at water first overtone region in addition to PC3.** (A, B) PC1 (A) and PC2 (B) score plots along the time time. (C, D) Their loadings (PC1 (C), PC2 (D)). The variations for PC1 and PC2 were 99.3717% and 0.6270%, respectively. For PC1, the change is score (closed circles) coincided well with that of scattering intensity (open triangles, see Figure 1), and it is therefore attributed mainly to change in the background intensities accompanying the fibrillation reaction. For PC2, although the loading pattern represented two peaks at 1409 and 1485 nm characteristic to the ion-hydrated and hydrogen-bonded (S_4_) waters, respectively, the time dependency seemed to be completed within 5 min, and we assigned this component not to changes in water structures accompanying the fibrillation process, but to those accompanying initial temperature jump of the sample solution. As a result, for PC1 and PC2, the change was attributed mainly to change in the light scattering accompanying the fibrillation reaction (A and C).
(TIF)

Figure S4 **PCA results of solvents (100 mM NaCl dissolved in 25 mM HCl) at water overtone region as shown for reference.** (A–C) PC1 (A), PC2 (B), and PC3 (C) score plots along the monitoring time. (D–F) Their loadings (PC1 (D), PC2 (E), PC3 (F)). The variations explained by PC1, PC2, and PC3 were 87.5405%, 12.4251%, and 0.0077%, respectively. For PC1, time dependency of score and its loading were almost the same as those of PC2 for the samples of fibrillation reaction (see Figure S3 B and D), and it is thus concluded to be assigned to changes in water structures accompanying initial temperature jump. PC2 score also showed a change completed within 5 min, which is plausibly caused by the temperature change, too. For PC3, although few negative peaks were found at around 1425 nm, the score fluctuated randomly throughout the measurement time period, which might trace random fluctuation of water structures inside the solvent and the remaining PC scores also showed random fluctuations throughout the measurement. Overall, no PCA score or loading similar to those observed for PC3 was found, supporting validity of the assignment of PC3 score and loading shown in Figure 4 to structural transformations of water molecules during the formation of insulin amyloid fibrils.
(TIF)

Figure S5 **PCA results of another dataset of protein solution analyzed at water region.** (A–C) PC1 (A), PC2 (B), and PC3 (C) score plots with monitoring time. (D–F) Their loadings (PC1 (D), PC2 (E), and PC3 (F)). The variations for PC1, PC2, and PC3 in the first dataset (black) were 99.3717%, 0.6270%, and 0.0005%, respectively, and those in the second dataset (red) were 99.5327%, 0.4661%, and 0.0003%, respectively. For PC1, the change in score coincided well with that of scattering intensity accompanying the fibrillation reaction. Based on this shape, three phases, i.e., phases I (nucleation), II (elongation), and III (equilibrium), were categorized which are colored by white, light gray, and dark gray, respectively in panel A and C. For PC2, although the loading pattern represented two peaks at 1409 and 1485 nm characteristic to ion-hydration and hydrogen-bonded (S_4_) waters, respectively, the time dependency seemed to be completed within 5 min, and we assigned this component not to changes in water structures accompanying the fibrillation process, but to those accompanying initial temperature jump of the sample solution. For PC3, the pattern of loading and score was quite similar with PC3 in the first measurement, and additionally the time-dependent changes in score coincided well with those of nucleation, elongation, and equilibrium phases, verifying the reproducibility of the characteristic transformations of water spectral patterns.
(TIF)

Figure S6 **The structural transformation of water molecules as estimated by PLS concentration regression analysis.** The analysis was performed with a dataset of NIR spectra in the region of 1300–1600 nm monitored at four different concentrations of insulin protein; 0.5, 1, 3, and 5 mg/ml. (A) Regression vector obtained as a model for the prediction of insulin concentration. The regression vector was based on the spectra at 6–10 min. The most prominent absorbance band was exhibited at around 1455 nm (assigned to S_2_), and additionally, positive peaks at 1365 nm (water solvation shell) and 1381 nm (OH^−^·(H_2_O)_5_ and/or O_2_^−^·(H_2_O)_4_), and negative peaks at 1421 nm (hydration), and 1437 nm (H^+^·(H_2_O)_2_) were observed, suggesting that hydrogen-bonded water structures are involved strongly with the formation of amyloid fibrils. (B) Time-dependent change in the SEP of protein concentration. In this analysis, the spectra at 6–10 min and 1–60 min were used for the model and test datasets, respectively. Although a markedly larger value at 1–5 min might indicate spectral change associated with temperature jump, the gradual increase in SEP values in the range of 11 min to 30 min coincides roughly with the elongation phase, verifying the change in amount of S_2_ water species. (C) Time-dependent change of the ratio of absorbance at 1460 nm (assigned to hydrogen-bonded water, S_2_) and that at 1410 nm (assigned to free water, S_0_). The result of protein solution is plotted by closed circles and that of solvent (25 mM HCl containing 100 mM NaCl) is also shown by open circles for reference. Immediately after starting the monitoring, because of the temperature increase and adjustment linked to increase of less hydrogen bonded water (S_0_), the overall ratio decreased. After 5 min, the value of absorbance ratio seemed to increased, which agreed very well with the observed 2 stages in the nucleation stage (Figure 4). Further on, in the next 2–3 minutes, it decreased very slightly and increased again showing details of the elongation phase (phase II). With this, we show that the changes in the initial stage of the fibril formation reaction are in accordance with the PCA and PLS regression results for protein concentration measurement.
(TIF)

Figure S7 **Time dependency of aquagram patterns of solvent (100 mM NaCl dissolved in 25 mM HCl) used in the present work.** (A) 6 min to 10 min for nucleation (corresponding to phase I in Figure 1). (B) 10 min to 18 min for elongation (phase II). (C) 18 min to 30 min for equilibrium phases (phase III). Aquagrams are plotted every 1 minute and those at 6 min, 10 min, 18 min, and 30 min are colored by blue, green, orange, and red, respectively. Although some amount of time dependency which plausibly indicates dynamics of water structures inside the solvent was observed, its patterns are distinct from those observed in protein samples in Figure 5. Based on this, it has been proposed that the pattern of aquagram, especially that observed in the nucleation phase, can be used as an indicator of fibril formation.
(TIF)
