# Supplementary figures and images for: Water Molecular System Dynamics Associated with Amyloidogenic Nucleation as Revealed by Real Time Near Infrared Spectroscopy and Aquaphotomics
Source: PLoS One. 2014 Jul 11;9(7):e101997. doi: 10.1371/journal.pone.0101997 (PMC4094474; doi:10.1371/journal.pone.0101997)

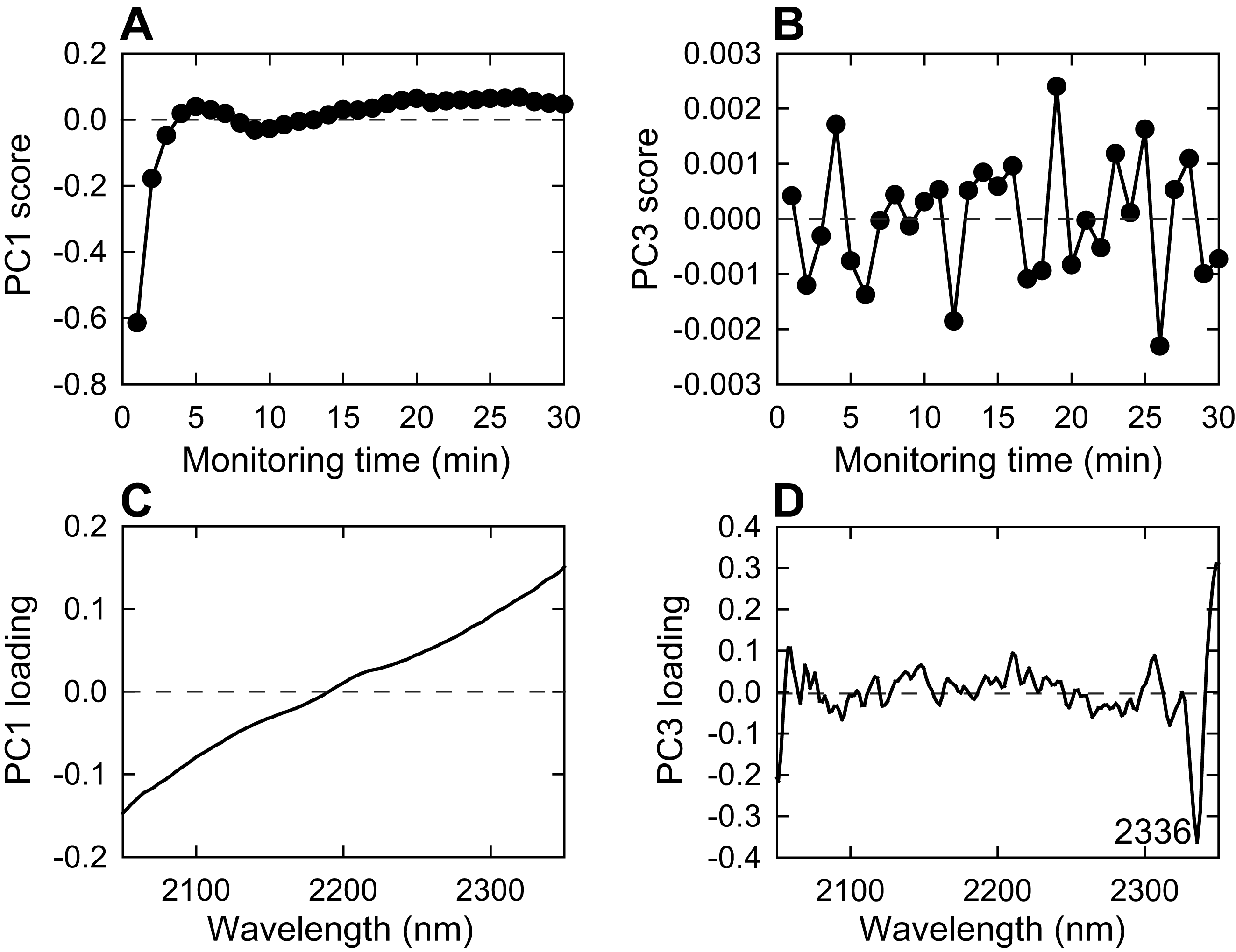

Supplement: Figure S1 — PC scores and loadings obtained as results of PCA at amide I overtone region in addition to PC2 (see Figure 2 ). (A, B) PC1 (A) and PC3 (B) score plots with monitoring time. (C, D) Their loadings (PC1 (C), PC3 (D)). The variations for PC1 and PC3 were 99.5362% and 0.0003%, respectively. For PC1, No characteristic peaks were observed and its time dependency seemed to be completed within 5 min, suggesting change in background intensities accompanying the initial temperature jump. For PC3, although one negative peak was found at 2336 nm, its score fluctuated randomly throughout the measurement time period and it was therefore difficult to find any correlations with nucleation or elongation phases of the fibril formation. (TIF) [file pone.0101997.s001.tif]

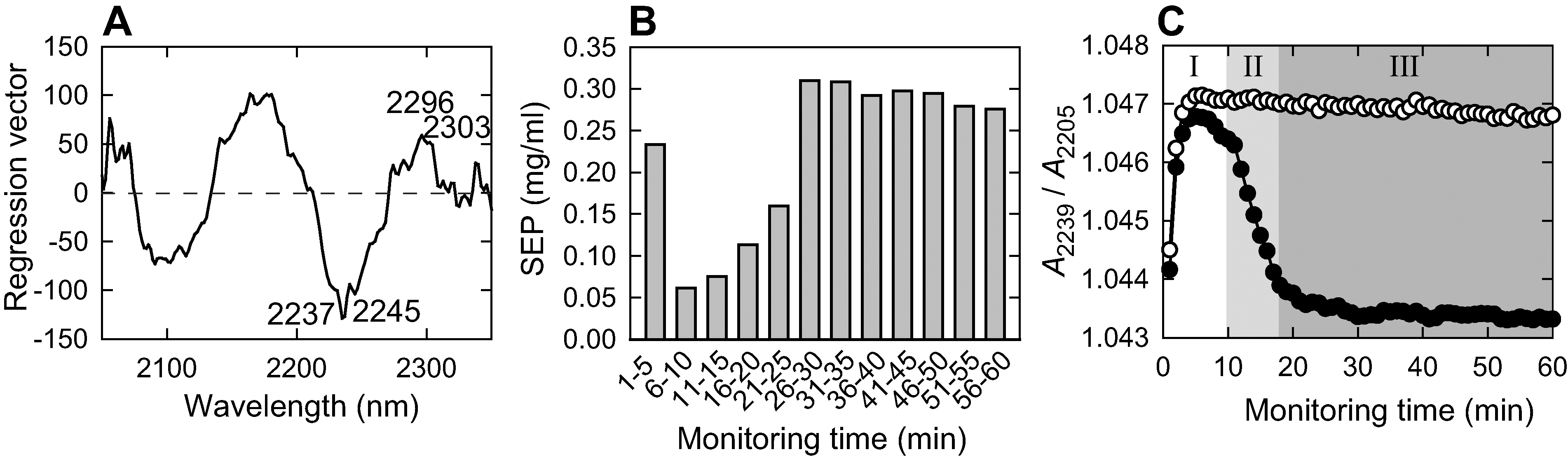

Supplement: Figure S2 — Spectral and structural changes of insulin protein molecules as estimated by PLS regression analysis. The analysis was performed with a dataset of NIR spectra over 2050–2350 nm monitored at four different concentrations of proteins; 0.5, 1, 3, and 5 mg/ml. (A) Regression vector obtained as a model for the prediction of insulin concentration. The regression vector was based on the spectra at 6–10 min. A negative and positive peak at around at 2240 nm (α-helix) and 2300 nm (β-sheet) respectively, were observed, suggesting that conformational transition from α-helix to β-sheet. (B) Time-dependent change in the standard error of prediction (SEP) of the insulin concentration. In this analysis, the spectra at 6–10 min and 1–60 min were used for the model and test datasets, respectively. Although a marked larger value at 1–5 min might indicate mainly spectral change associated with temperature jump, the gradual increase in SEP values in the range of 11 min to 30 min coincides roughly with the elongation phase, verifying the formation of β-sheet rich fibril structure. (C) Time-dependent change of the ratio of absorbance at 2239 nm (assigned to α-helix) and that at 2205 nm (assigned to β-sheet). The result of protein solution is plotted by closed circles and that of solvent (25 mM HCl containing 100 mM NaCl) is also shown by open circles for reference. A marked decrease of the value in elongation (phase II) was observed, suggesting the abrupt increase in cross-β structure after undergoing the lag phase for nucleation, in accordance with the PLS result. (TIF) [file pone.0101997.s002.tif]

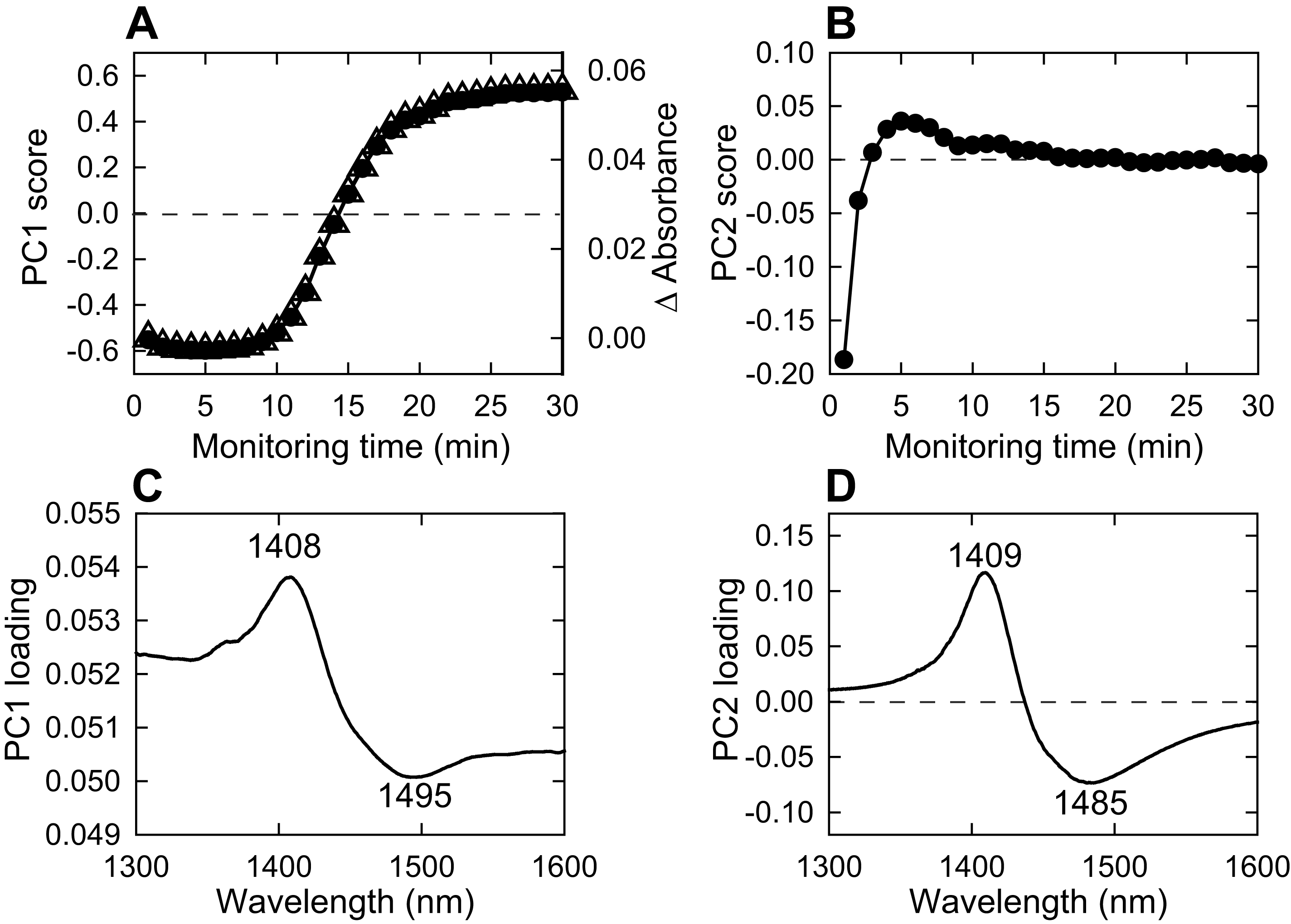

Supplement: Figure S3 — Information of PC scores and loadings obtained as results of PCA at water first overtone region in addition to PC3. (A, B) PC1 (A) and PC2 (B) score plots along the time time. (C, D) Their loadings (PC1 (C), PC2 (D)). The variations for PC1 and PC2 were 99.3717% and 0.6270%, respectively. For PC1, the change is score (closed circles) coincided well with that of scattering intensity (open triangles, see Figure 1), and it is therefore attributed mainly to change in the background intensities accompanying the fibrillation reaction. For PC2, although the loading pattern represented two peaks at 1409 and 1485 nm characteristic to the ion-hydrated and hydrogen-bonded (S4) waters, respectively, the time dependency seemed to be completed within 5 min, and we assigned this component not to changes in water structures accompanying the fibrillation process, but to those accompanying initial temperature jump of the sample solution. As a result, for PC1 and PC2, the change was attributed mainly to change in the light scattering accompanying the fibrillation reaction (A and C). (TIF) [file pone.0101997.s003.tif]

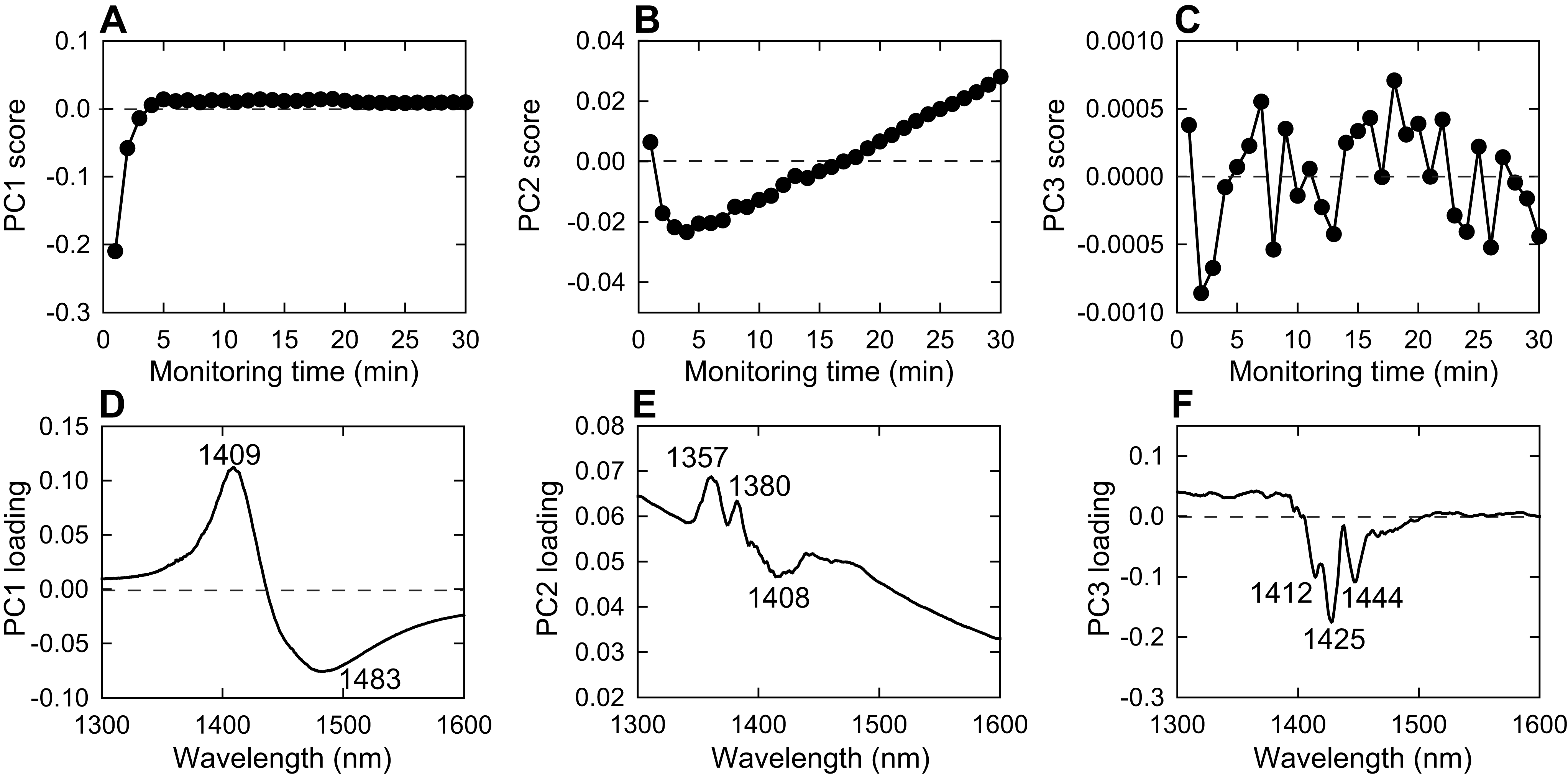

Supplement: Figure S4 — PCA results of solvents (100 mM NaCl dissolved in 25 mM HCl) at water overtone region as shown for reference. (A–C) PC1 (A), PC2 (B), and PC3 (C) score plots along the monitoring time. (D–F) Their loadings (PC1 (D), PC2 (E), PC3 (F)). The variations explained by PC1, PC2, and PC3 were 87.5405%, 12.4251%, and 0.0077%, respectively. For PC1, time dependency of score and its loading were almost the same as those of PC2 for the samples of fibrillation reaction (see Figure S3 B and D), and it is thus concluded to be assigned to changes in water structures accompanying initial temperature jump. PC2 score also showed a change completed within 5 min, which is plausibly caused by the temperature change, too. For PC3, although few negative peaks were found at around 1425 nm, the score fluctuated randomly throughout the measurement time period, which might trace random fluctuation of water structures inside the solvent and the remaining PC scores also showed random fluctuations throughout the measurement. Overall, no PCA score or loading similar to those observed for PC3 was found, supporting validity of the assignment of PC3 score and loading shown in Figure 4 to structural transformations of water molecules during the formation of insulin amyloid fibrils. (TIF) [file pone.0101997.s004.tif]

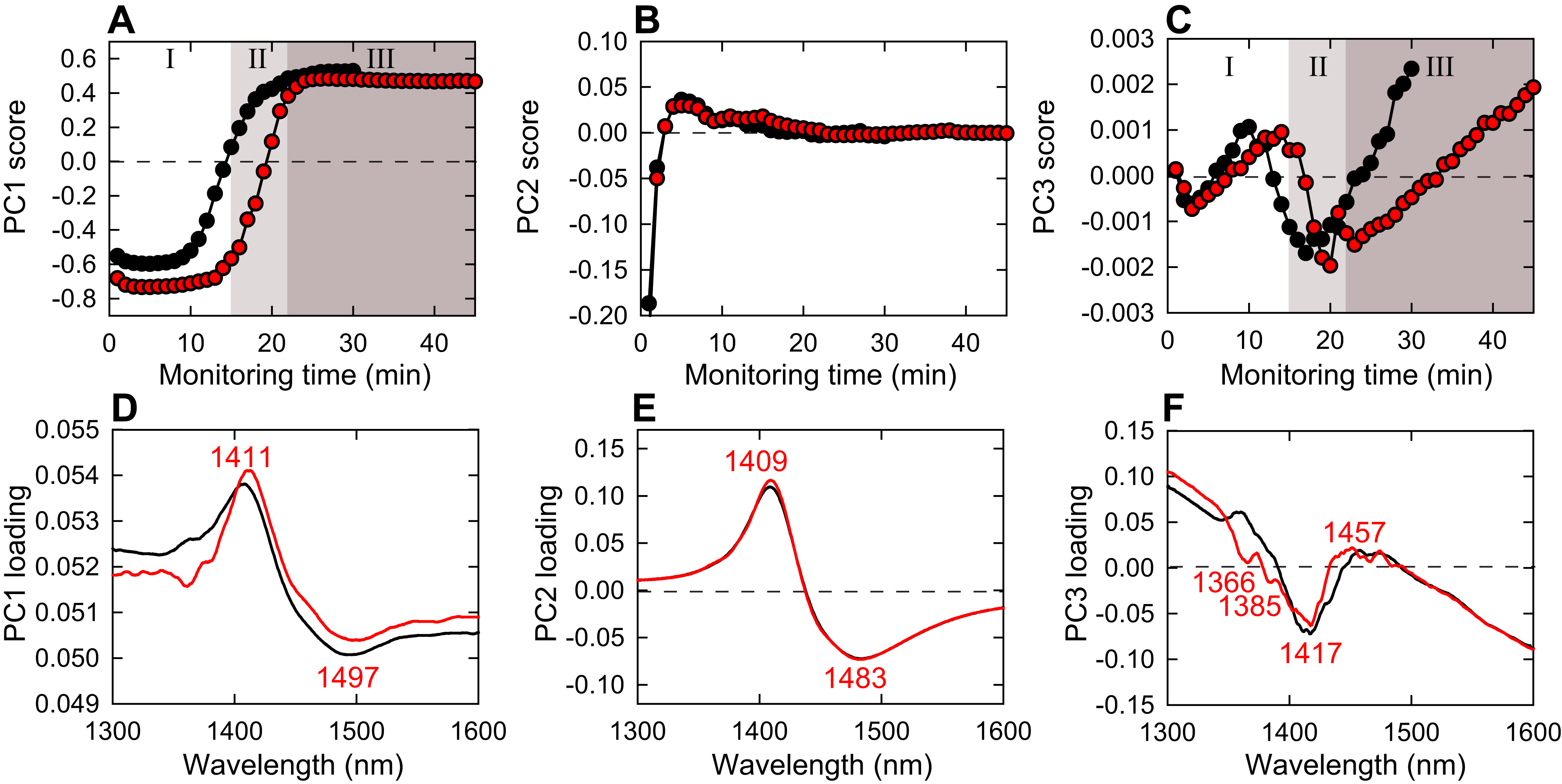

Supplement: Figure S5 — PCA results of another dataset of protein solution analyzed at water region. (A–C) PC1 (A), PC2 (B), and PC3 (C) score plots with monitoring time. (D–F) Their loadings (PC1 (D), PC2 (E), and PC3 (F)). The variations for PC1, PC2, and PC3 in the first dataset (black) were 99.3717%, 0.6270%, and 0.0005%, respectively, and those in the second dataset (red) were 99.5327%, 0.4661%, and 0.0003%, respectively. For PC1, the change in score coincided well with that of scattering intensity accompanying the fibrillation reaction. Based on this shape, three phases, i.e., phases I (nucleation), II (elongation), and III (equilibrium), were categorized which are colored by white, light gray, and dark gray, respectively in panel A and C. For PC2, although the loading pattern represented two peaks at 1409 and 1485 nm characteristic to ion-hydration and hydrogen-bonded (S4) waters, respectively, the time dependency seemed to be completed within 5 min, and we assigned this component not to changes in water structures accompanying the fibrillation process, but to those accompanying initial temperature jump of the sample solution. For PC3, the pattern of loading and score was quite similar with PC3 in the first measurement, and additionally the time-dependent changes in score coincided well with those of nucleation, elongation, and equilibrium phases, verifying the reproducibility of the characteristic transformations of water spectral patterns. (TIF) [file pone.0101997.s005.tif]

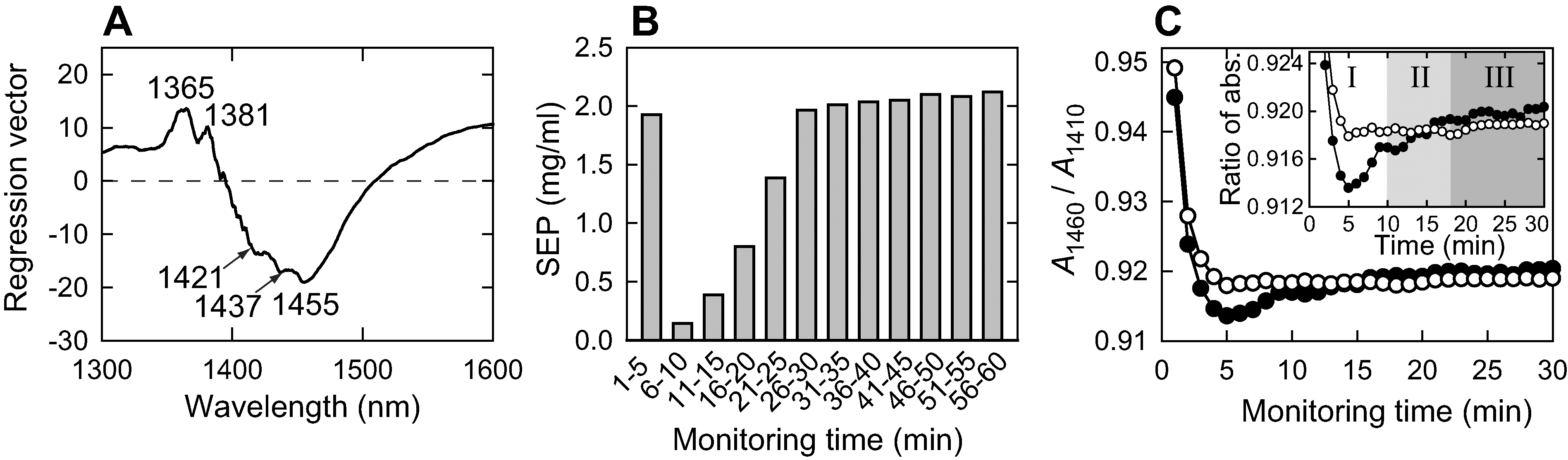

Supplement: Figure S6 — The structural transformation of water molecules as estimated by PLS concentration regression analysis. The analysis was performed with a dataset of NIR spectra in the region of 1300–1600 nm monitored at four different concentrations of insulin protein; 0.5, 1, 3, and 5 mg/ml. (A) Regression vector obtained as a model for the prediction of insulin concentration. The regression vector was based on the spectra at 6–10 min. The most prominent absorbance band was exhibited at around 1455 nm (assigned to S2), and additionally, positive peaks at 1365 nm (water solvation shell) and 1381 nm (OH−·(H2O)5 and/or O2 −·(H2O)4), and negative peaks at 1421 nm (hydration), and 1437 nm (H+·(H2O)2) were observed, suggesting that hydrogen-bonded water structures are involved strongly with the formation of amyloid fibrils. (B) Time-dependent change in the SEP of protein concentration. In this analysis, the spectra at 6–10 min and 1–60 min were used for the model and test datasets, respectively. Although a markedly larger value at 1–5 min might indicate spectral change associated with temperature jump, the gradual increase in SEP values in the range of 11 min to 30 min coincides roughly with the elongation phase, verifying the change in amount of S2 water species. (C) Time-dependent change of the ratio of absorbance at 1460 nm (assigned to hydrogen-bonded water, S2) and that at 1410 nm (assigned to free water, S0). The result of protein solution is plotted by closed circles and that of solvent (25 mM HCl containing 100 mM NaCl) is also shown by open circles for reference. Immediately after starting the monitoring, because of the temperature increase and adjustment linked to increase of less hydrogen bonded water (S0), the overall ratio decreased. After 5 min, the value of absorbance ratio seemed to increased, which agreed very well with the observed 2 stages in the nucleation stage (Figure 4). Further on, in the next 2–3 minutes, it decreased very slightly and increased again showing de [file pone.0101997.s006.tif]

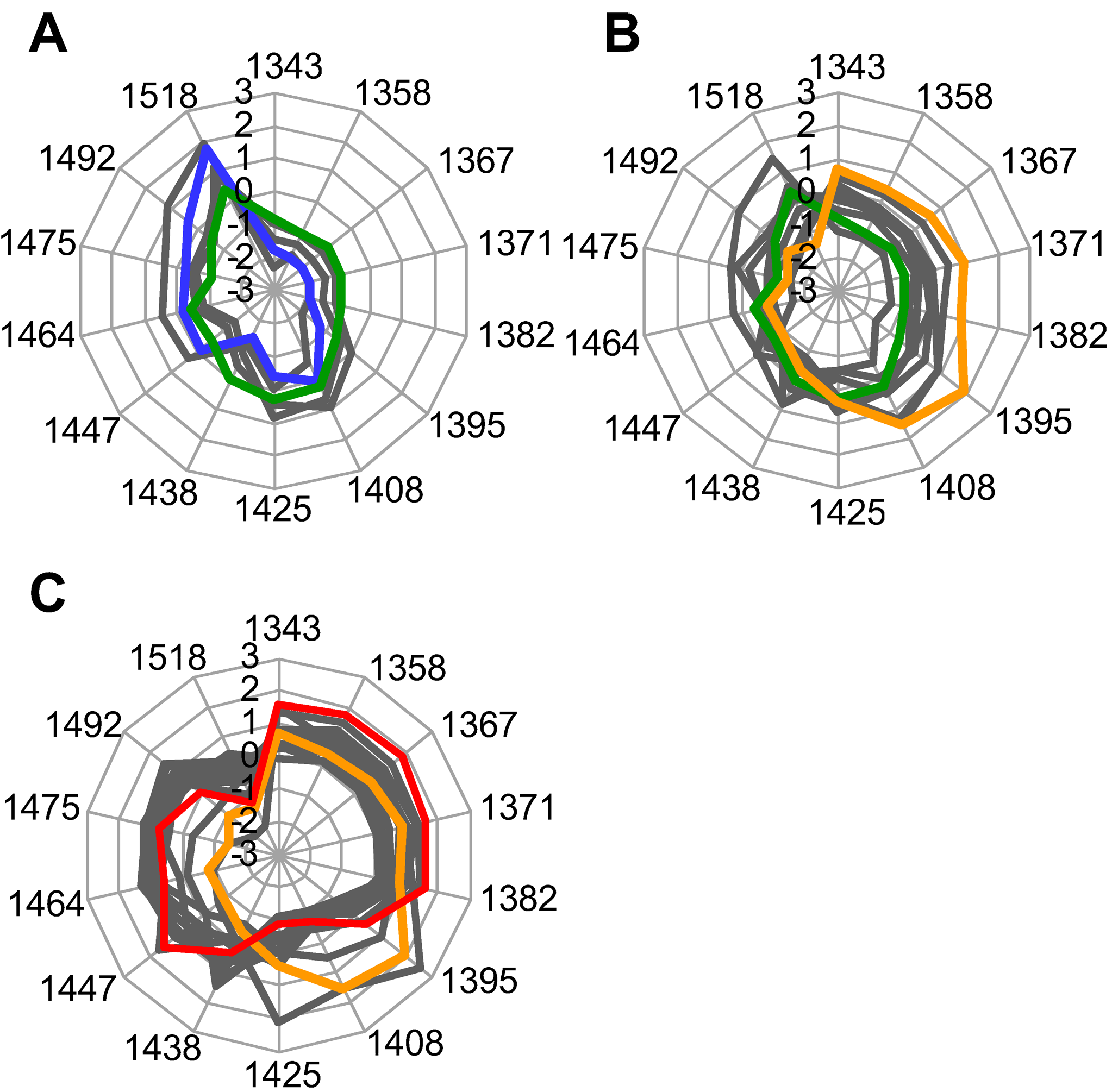

Supplement: Figure S7 — Time dependency of aquagram patterns of solvent (100 mM NaCl dissolved in 25 mM HCl) used in the present work. (A) 6 min to 10 min for nucleation (corresponding to phase I in Figure 1). (B) 10 min to 18 min for elongation (phase II). (C) 18 min to 30 min for equilibrium phases (phase III). Aquagrams are plotted every 1 minute and those at 6 min, 10 min, 18 min, and 30 min are colored by blue, green, orange, and red, respectively. Although some amount of time dependency which plausibly indicates dynamics of water structures inside the solvent was observed, its patterns are distinct from those observed in protein samples in Figure 5. Based on this, it has been proposed that the pattern of aquagram, especially that observed in the nucleation phase, can be used as an indicator of fibril formation. (TIF) [file pone.0101997.s007.tif]
